# Stress regulated members of the plant organic cation transporter family are localized to the vacuolar membrane

**DOI:** 10.1186/1756-0500-1-43

**Published:** 2008-07-11

**Authors:** Isabell Küfner, Wolfgang Koch

**Affiliations:** 1 Center for Plant Molecular Biology-Plant Biochemistry, Auf der Morgenstelle 5, 72076 Tübingen, Germany; 2 Center for Plant Molecular Biology-Plant Physiology, Auf der Morgenstelle 1, 72076 Tübingen, Germany

## Abstract

**Background:**

In Arabidopsis six genes group into the gene family of the organic cation transporters (OCTs). In animals the members of the OCT-family are mostly characterized as polyspecific transporters involved in the homeostasis of solutes, the transport of monoamine neurotransmitters and the transport of choline and carnitine. In plants little is known about function, localisation and regulation of this gene family. Only one protein has been characterized as a carnitine transporter at the plasma membrane so far.

**Findings:**

We localized the five uncharacterized members of the Arabidopsis OCT family, designated OCT2-OCT6, via GFP fusions and protoplast transformation to the tonoplast. Expression analysis with RNA Gel Blots showed a distinct, organ-specific expression pattern of the individual genes. With reporter gene fusion of four members we analyzed the tissue specific distribution of OCT2, 3, 4, and 6. In experiments with salt, drought and cold stress, we could show that *AtOCT4, 5 *and *6 *are up-regulated during drought stress, *AtOCT3 *and *5 *during cold stress and *AtOCT 5 *and *6 *during salt stress treatments.

**Conclusion:**

Localisation of the proteins at the tonoplast and regulation of the gene expression under stress conditions suggests a specific role for the transporters in plant adaptation to environmental stress.

## Findings

### Background

Controlled transport processes across membranes of different compartments and between organs are essential for plant nutrient and ion distribution. Changing environmental conditions, like altered nutrient availability, water supply and temperature, require adequate responses by the plant in primary and secondary metabolism. The plant vacuole is a central compartment for this complex process of adaptation to altered environmental conditions. The transport of ions and solutes across the tonoplast is a rapid way to adjust and maintain the required concentrations in the cytoplasm and to avoid the accumulation of toxic substances or ion concentrations [1-3]. For such transport processes several substrate specific membrane proteins have been identified at the tonoplast. These are examples for transporters for water and organic solutes, like urea, sugars and sugar alcohols, as well as transporters for monovalent and divalent inorganic cations (summarized in [4]). But, compared to the high number and variation of primary and secondary metabolites found in the vacuole, the number of transporters identified at the tonoplast is relatively low.

### The SLC22 family and plant OCTs

The human solute carrier family 22, (SLC 22), is a gene family that contains organic cation transporters (OCTs), zwitterions/cation transporters (OCTNs) and organic anion transporters (OATs,) with 11–12 transmembrane domains. They can function as uniporters (OCTs), cotransporters (OCTN2) or anion exchangers (for review see [5]). In most cases these transporters are polyspecific and shuttle various substrates e.g. monoamine neurotransmitters, choline, uric acid and prostaglandine, but also α-ketoglutarate and carnitine. In plants, the first protein related to the SLC22 family was identified in *Phaseolus vulgaris, PvOCT1 *[6]. While the substrate of *PvOCT1 *is unclear, it was assumed that it plays a role in stress adaption, as its expression is up-regulated after drought stress. However, the Arabidopsis homolog of *PvOCT1*, AtOCT1, has been characterized functionally as a carnitine transporter at the plasma membrane [7]. No information about the role, the subcellular localization or the gene regulation of the other five members in Arabidopsis is available up to now. In a recent proteomic approach of isolated vacuoles from cauliflower, a homolog to the Arabidopsis protein At1g16390 (AtOCT3) was found. The localization of the protein at the tonoplast was verified via transient expression of a GFP fusion in protoplasts [8]

## Results

### Subcellular localization of the AtOCTs

**Figure 1:**
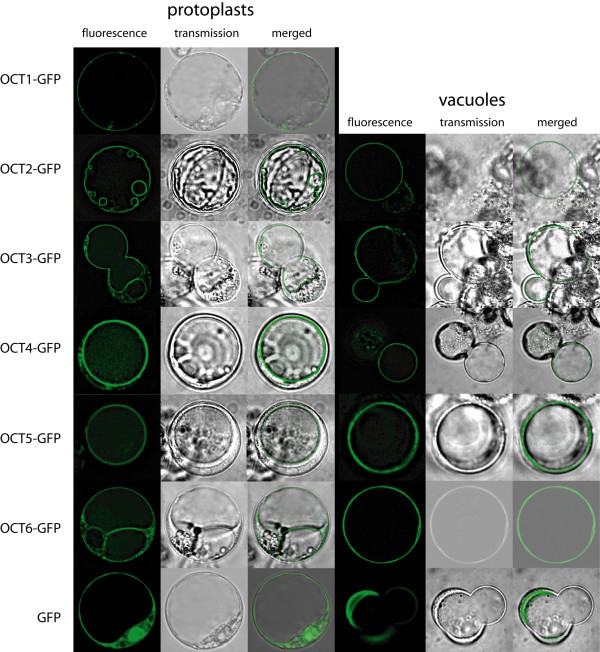
**Subcellular localization of AtOCT1-AtOCT6 using cDNA-GFP fusions and protoplast transformation**. Left part: Subcellular localisation studies of AtOCT-GFP fusion proteins. OCT proteins were fused with GFP at the C-terminus and expressed transitionally in protoplasts of Arabidopsis Col-0 cell cultures. Protoplasts expressing the GFP protein under the control of the CaMV 35S promoter were used as control. GFP fluorescence was detected throughout the cytosol. The GFP fluorescence (left panel) and the bright field image (middle panel) were overlayed in the right panel. AtOCT1-GFP fluorescence was detected at the plasma membrane as shown before [7]. Fusion proteins of AtOCT2-AtOCT6-GFP were localized at membranes in the protoplasts, most probably at the tonoplast. Right part: To confirm the assumed vacuolar localisation, vacuoles were released by an osmotic shock from the protoplasts. GFP signal of the fusion proteins of AtOCT2-AtOCT6-GFP is detected at the free vacuole or at vacuoles just escaping from the protoplasts. Free GFP signal is localised to the cytosol.

In silico analysis of the AtOCT protein sequences predicted membrane proteins with 11–13 transmembrane domains. The protein structures of five AtOCTs are quite similar, whereas the structure of the plasmamembrane localized AtOCT1 differs in the N-terminal region (additional file 1: Consensus prediction of transmembrane domains of the AtOCTs). However, the subcellular distribution of the proteins is still unclear although several prediction programmes, like aramemnon [9] were used. To discover the subcellular distribution we fused the open reading frames of all the family members translationally to GFP and expressed the proteins transiently in protoplasts. As a control, AtOCT1 was used for localization to the plasma membrane as shown before [7]. As expected, AtOCT1 ended up in the plasmamembrane, whereas all the other five members (AtOCT2-6) localized to intracellular membranes, most likely the tonoplast (Figure 1, left part). For verification of the tonoplast localisation an osmotic shock was applied to release the vacuoles from the protoplasts. In the right part of figure 1, tonoplast pictures or tonoplasts escaping from the vacuoles are shown. For all five fusion proteins of AtOCT2-6-GFP the signal is associated with the tonoplast.

### Organ and tissue specific expression of the *AtOCTs*

RNA-gel blot analysis showed an expression of the five vacuolar *AtOCTs *in a distinct organ-specific manner (additional file 2: Organ specific expression *AtOCT2-AtOCT6*). *AtOCT2 *is in genera lweakly expressed. *AtOCT3 *mRNA was only present in siliques, but here in a higher amount. Expression of *AtOCT4 *mRNA was strongest in roots and also showed a very faint background expression in the other organs. *AtOCT5 *expression is strongest in sink leaves and source leaves. Together with *AtOCT4, AtOCT6 *was the second gene expressed predominantly in roots and weakly in the stem.

To analyze the tissue specific expression of the *AtOCTs*, promoter GUS fusions for AtOCT2,3,4 and 6 were constructed and transformed in Arabidopsis. P_*AtOCT*2_-GUS-expression in flowers was restricted to pollen grains and the stigma (additional file 3: GUS activitiy under control of the *AtOCT2 *Promoter). GUS activity was detected in the vascular tissue of older siliques and also in the envelope of young siliques (C, D). Staining of the whole rosette revealed a leaf age-dependent expression pattern (F). In young leaves the whole leaf blade except the vasculature was stained (H,I,J). Cross sections showed that the staining is located in the upper epidermis and the cell layer below (I). At the inner part of young leaves the staining is in the parenchyma cells below the vasculature (J). In mature leaves P_AtOCT2_-GUS expression was only detectable in the phloem (G,K). In roots expression located to the two vascular strands, the initiation site of lateral roots and at the root tip (L,M,N).

**Figure 2:**
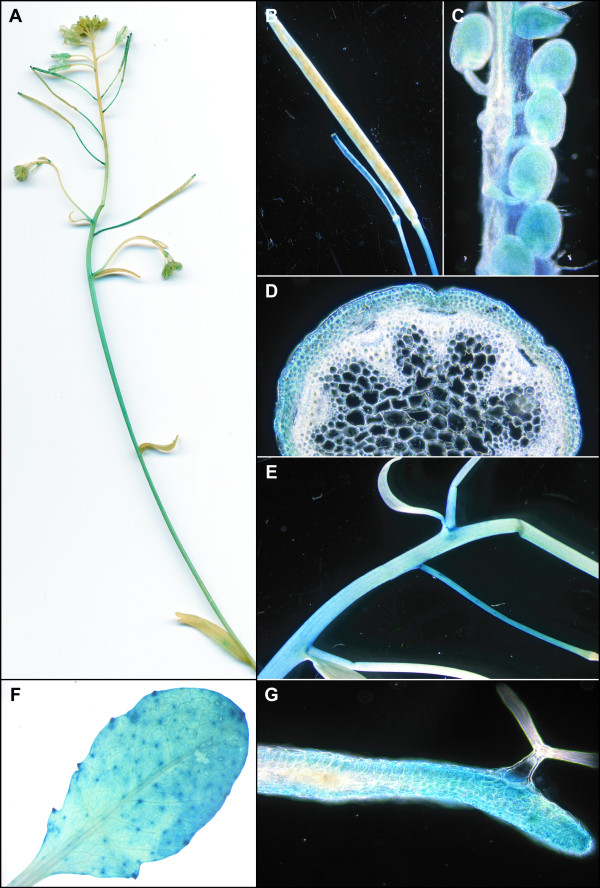
**Tissue-Specific GUS Activity under the Control of the *AtOCT3 *Promoter**. P_OCT3_-Gus plants show staining in the basal part of the stem (A) and in seeds in young siliques only (B,C) but not in mature seeds (C). Cross section of the stem localized the staining to the epidermis and parenchyma cells below the epidermis (D). The stems of secondary inflorescence show strong staining (E). In leaves, staining was visible throughout the leaf blade and the basis of trichomes (F), cross section of a leaf shows GUS signal at lower and upper epidermis (G).

**Figure 3:**
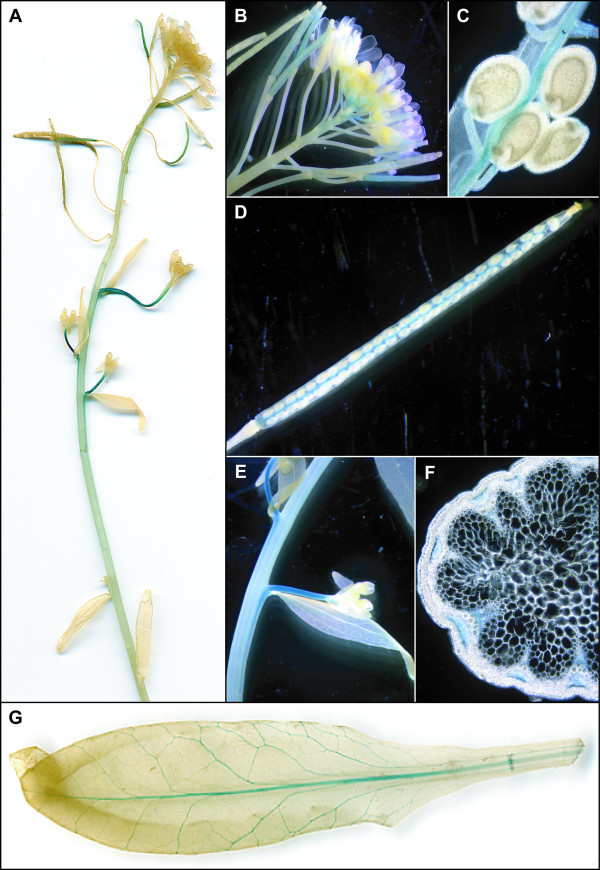
**Tissue-Specific GUS Activity under the Control of the *AtOCT4 *Promoter**. In the stem of P_AtOCT4_-plantsGus activity was weak, a stronger signal was present in the stem of secondary inflorescences (A, E). In the flowers, GUS activity can be found in young siliques (B). Opening of the siliques reveals that the staining is restricted to the veins of young (C) and mature siliques (D). Cross sections of the stem localize the staining to the phloem and cells medial to the xylem (F). In rosette leaves GUS activity can be found in the vasculature (G).

**Figure 4:**
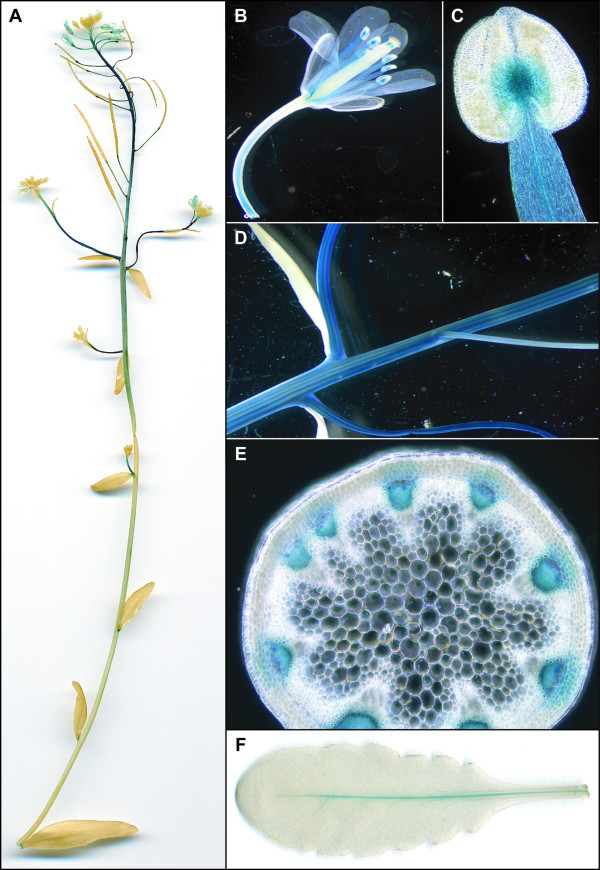
**Tissue-Specific GUS Activity under the control of the *AtOCT6 *Promoter**. An overview of the stem shows staining of secondary inflorescences and flowers (A). Close up reveals GUS activity in the filaments and the tip of the filaments and the connective tissue (B, C). Close up of the stem shows stripes along the stem and a cross section localizes the GUS staining to the phloem (E). In rosette leaves GUS activity is restricted to the major vein (F).

In P_AtOCT3_-GUS plants, expression in the siliques was mainly found in young seeds but not in the envelope or in older seeds (Figure 2B,C). Strong GUS activity was visible in the stem, and cross sections localized the staining to cortical cells and parenchyma cells below the cortex (Figure 2A,D). Furthermore, GUS activity was also high at the basis of secondary inflorescences (Figure 2E). In leaves, expression was found in the lower and upper epidermis and at the base of the trichomes (Figure 2F,G). Similarly to *AtOCT2*, expression of P_AtOCT4-_GUS was detected in young siliques and the vascular tissue of the siliques, and not in the seeds (Figure 3A,B,C,D). A strong signal of P_AtOCT4-_GUS was also detected in the stems of secondary inflorescences (Figure 3A,E), which (in cross-sections) appears to be in the phloem cells and xylem parenchyma cells (Figure 3F). Related to P_AtOCT4_-GUS plants, for P_AtOCT6_-GUS plants, a strong staining in plants was detected in the stem of secondary inflorescences (Figure 4A,D) which could be localized to the phloem (Figure 4E). In flowers, P_AtOCT6_-*GUS *expression was found in the stamen, in the filaments and the connective (Figure 4B,C). In rosette leaves, expression is restricted to the major veins of mature leaves (Figure 4F).

### Stress specific induced expression of *AtOCTs*

**Figure 5:**
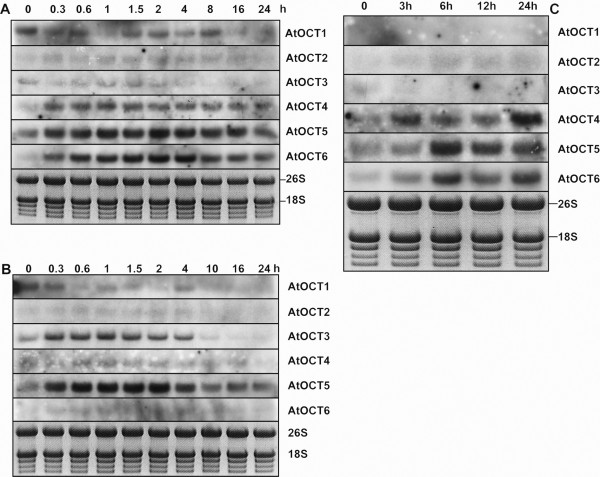
**Specific expression of vacuolar *AtOCTs *during drought, cold and salt stress**. A) Expression of *AtOCTs *in response to drought stress. Expression was monitored in seedlings at indicated time points by RNA-Gel-blot analysis. 20 minutes after initiation of desiccation transcripts of *AtOCT4 5*and *6 *start to accumulate over a period of 8 hours, with a maximum around 2–4 hours. *AtOCT5 *and *AtOCt6 *respond with a strong accumulation of transcripts whereas *AtOCT2 *and *AtOCT3 *do not respond at all. B) Expression of *AtOCTs *in response to cold stress. Expression was monitored in seedlings at indicated time points by RNA-Gel-blot analysis after transfer of seedling to 4°C. Increased levels of transcript were found 20 minutes after induction for *AtOCT3 *and *AtOCT5 *with a transient increase over 2 hours and decreasing after 4 hours. *AtOCT2*, *4 *and *6 *do not respond to cold stress. C) Expression of *AtOCTs *in respond to salt stress. Transcripts of *AtOCT4, 5 *and *6 *respond to salt stress, *AtOCT5 *and *AtOC6 *expression shows a maximum level of transcripts after *6 *hours and *AtOCT4 *after 24 hours. The expression of *AtOCT2 *and *3 *does not respond to salt stress.

Since the vacuole is involved in many processes related to osmoregulation and homeostasis of solutes, we tested whether the expression of the *AtOCTs *responds to stress situations that are known to affect the homeostasis of the cytosol. Cold, drought and salt stress induce processes that involve the vacuole as an important buffering "tank". In the stress experiments we could show that the vacuolar AtOCTs respond differentially with enhanced expression in timing and strength to the treatments. Drought stress causes a rapid increase of *AtOCT4, 5 *and *6 *transcripts within 20 minutes of the initiation of desiccation (Figure 5A). A maximum expression level was reached ~2 h after the start of treatment. *AtOCT2 *and *3 *gene expression was not induced after drought. Cold treatment of the seedlings resulted in an increased expression level of *AtOCT3 *and *5 *again within 20 minutes (Figure 5B). *AtOCT2, 4 *and *6 *do not respond to cold stress treatment. In the salt stress experiment timing of the induction is slower but here *AtOCT5 *and *AtOCT6 *respond with increased expression levels six hours after exposure to salinity (Figure 5C). *AtOCT2 *and *3 *do not respond and *AtOCT4 *expression is increased after 3 h and decreases with a second peak after 24 h. In all three experiments *AtOCT5 *expression responds with the strongest increase of transcript levels. *AtOCT3 *expression is only increased during cold stress treatment. The expression of *AtOCT1*, the plasmamembrane localized member of this family, is not induced after the stress treatments, but rather reduced upon drought and salt stress.

## Conclusion

The five vacuolar membrane proteins of the AtOCT family show a distinct individual response to drought, cold and salt stress. The only plasmamembrane member of this gene family does not respond to these stresses. Discrepancies to already published expression data [10], where drought induction of AtOCTs is weak derive most likely from the different experimental procedure. Here we used permanent drought stress whereas Kilian et al. used a 15 minutes drought stress and then reapplied water [11]. The substrate specificity of the animal OCTs is not very high, which might also be the case for the plant vacuolar OCTs. Polyspecificity would allow the plants to react rapidly and efficiently with the up regulation of transporters as an answer to the multiple compounds that accumulate under various stress conditions [12-14]. Either the AtOCTs could detoxify these compounds into the vacuole or release compatible solutes stored in the tonoplast. Expression in the phloem could indicate an enhanced content of compatible solutes in the phloem cells as a response to salt stress or water deficit as described for proline and sugar alcohols [15,16]. The potentially complex substrate spectrum might make it difficult to address the individual substrates for the proteins. Based on the tissue specific expression of the genes and on the stress induction data, a metabolic analysis of T-DNA insertion lines will help in identifying potential substrates.

## Methods

### Plant Material

*Arabidopsis thaliana *L. ecotype Col-0 was either grown in axenic culture on MS medium [17] supplemented with 2% sucrose or cultured in soil in the greenhouse. Arabidopsis plants were transformed using *Agrobacterium tumefaciens *pGV3101 under vacuum infiltration as described [18]. Cold stress: Plants were grown for 3 weeks in sterile culture on MS plates (14 h light, 21°C) and then transferred to 4°C at the beginning of the light period. Samples were harvested and frozen in liquid nitrogen prior to RNA-extraction. Drought stress: Plants were grown as above and transferred from petridishes to filter paper (whatman 3 MM). For salt stress experiments plants were grown hydroponically as described before [19] and NaCl concentration was adjusted to 200 mM at the start of the experiments.

### RNA-Work

Total RNA was isolated from seedlings, mature leaves, stems and other organs with phenol following LiCl precipitation, separation and transfer to nylon membranes as described [20]. Labeling with α^32^P-dATP was performed with Hexalabel DNA labeling Kit (MBI, Fermentas). Hybridization was performed at 65°C in 0.25 M sodium phosphate pH 7.2, 7% SDS, 1 mM EDTA and 1% BSA for 16 h using the cDNAs of AtOCTs and actin as a probe. Filters were washed twice with 2 × SSC/0.1% SDS and 0.2 × SSC/0.1% SDS for 20 min at 65°C and exposed to X-ray films

### DNA-Work

#### Green fluorescent Protein (GFP) fusion

The RT-PCR amplified ORFs of *AtOCTs *were cloned behind the CaMV 35S promoter in front to GFP5 (S65T). Restricition sites used were for *AtOCT1(At1g73220), AtOCT2(At1g79360)*, *AtOCT5(At1g79410) SacI*/*Bam*HI, for *AtOCT6(At1g16370)*, *AtOCT3(At1g16390) Bam*HI/*Bsp*HI and *AtOCAT4 (At3g20660) KpnI*/*Bsp*HI, The linker between the AtOCTs and GFP was 7–8 amino acids (WGIQGDII for AtOCT1, AtOCT2, and AtOCT5, WGAGAGV for AtOCT6 and AtOCT3 and YGAGAGVfor AtOCT4). The primers used were AtOCT1 ATG/*SacI *5'-ggggagctcATGGAACCTTCAAAACAAGAAG-3', AtOCT1 *Bam*HI 5'-cccggatccccCAAGTAATCATGATTGTTTCG-3', AtOCT2 ATG/*SacI *5'-aaagagctcATGGCAGAACCAACTCAG-3', AtOCT2 *Bam*HI 5'-cccggatccccCATGCAATGACATTATTAACG-3, AtOCT3 ATG/*Bam*HI 5'-tttggatccATGGCCGACTCGACTCG-3, AtOCT3 *Bsp*HI 5'-cctcatgactcctgcgccagcacccCAACCAATAAATTGTCTTTTTGC-3', AtOCT4 ATG/*KpnI *5'-aaaaaggtaccATGGAATCTCCGGAGGATAG-3, AtOCT4 *Bsp*HI 5'-ccctcatgactcctgcgccagcaccaTAACATATTACTTCTCCTCTTTC-3, AtOCT5 ATG/*SacI 5'-*tttgagctcATGGCGGATTCGTTGGC-3, AOCT5 *Bam*HI 5'-cccggatccccCAGCAACTATGGCTAGTC-3' AtOCT6 ATG/*Bam*HI 5'-tttggatccATGGCTGATCCAATATCAG-3', AtOCT6 *Bsp*HI 5'-aaatcatgactcctgcgccagcacccCAGCAAACATGGCTGG-3',

#### Promoter glucuronidase (GUS) fusion

Promoter GUS construct consist of promoter including first 21–24 bases of the ORFs and were fused translationally to GUS. The fragments were cloned in pBluescript SK (-) (Stratagene, La Jolla, USA) confirmed by sequencing. Subsequently the total promoter constructs were cloned in frame to *uidA *(GUS) of pCB308 [21]. The length of the fragments were: P_OCT2-_2210 bp, P_OCT3_-1553 bp, P_OCT4_-900 bp, P_OCT5_-1873 bp, P_OCT6_-1683 bp, and the following primers with restriction sites were used.

P-GUS OCT2f *Bcu*I 5'-gggactagtTACCTCTGCTCAGTTGG-3'

P-GUS OCT2r *Sma*I 5'-gggAGCGGCTGAGTTGGTTCTG-3'

P-GUS OCT3f *Bcu*I 5'-gggactagtTTTCTTGATTCGATTTTGAGC-3'

P-GUS OCT3r *Sma*I 5'-gggAGAAGCGGCCGAGTCGAGTC-3'

P-GUS OCT4f *Bcu*I 5'-gggactagtAAGCGTAAGAGGACGCTC-3'

P-GUS OCT4r *Sma*I 5'-gggTTTCTATCCTCCGGAGATTCC-3'

P-GUS OCT5f *Bam*HI 5'-gggggatccGATGTATATGTGTGTAGAGAGAG-3'

P-GUS OCT5r *Sma*I 5'-gggGCCATGGTTGCTTACTTTGATCG-3'

P-GUS OCT6f *Bcu*I 5'-gggactagtTTTGGAGTAAGAATTGGTTTG-3'

P-GUS OCT6r *Sma*I 5'-gggGGTTCTGATATTGGATCAGCC-3'

### Protoplast-Work

Transient transformation of the protoplasts with polyethylene glycol was performed according to the protocol of Negrutiu et al [22]. Transient GFP expression was monitored 24 h after transformation. Vacuoles were released from protoplast by creating an osmotic shock by adding water (1:1 to the protoplast suspension) and escaping vacuoles were monitored immediately.

### Histochemical localization of GUS activity

Histochemical assays for β-glucuronidase activity were performed as previously described. [23]. Briefly, for the fresh sections, tissues were embedded in 5% low melting agarose, and agar blocks were cut (75 – 150 μm) with razor blades using a vibratome (Leica, Germany).

## Competing interests

The authors declare that they have no competing interests.

## Authors' contributions

IK carried out the work and designed the experiments together with WK. WK wrote the manuscript and managed the overall project. Both authors read and approved the final manuscript.

## Supplementary Material

Additional File 1 **Consensus prediction of transmembrane domains of the AtOCTs**. Hydropathie analysis using the ARAMEMNON database [34] is based on the consensus of 11 prediction programmes. For AtOCT1 and AtOCT6 13 transmembrane domains are predicated, for AtOCT2, AtOCT3, and AtOCT4 12 transmembrane domains and for AtOCT5 11. Numbers score the probability of each predicted membrane domain of the AtOCTs. Click here for file

Additional File 2 **Organ specific expression *AtOCT2-AtOCT6 *using RNA-Gel blot analysis**. RNA-gel blot analysis showed a specific expression of the individual AtOCTs in Arabidopsis. *AtOCT2 *revealed a weak expression in flowers and siliques, *AtOCT4 *and *AtOCT6 *showed a predominant expression in roots, and *AtOCT6 *is also expressed in the stem. *AtOCT 5 *expression was strongest in sink leaves and weaker in mature leaves. *AtOCT3 *expression is strongest in siliques and a weak signal is detected in flowers, *AtOCT2,3*, and *5 *also showed a weak signal in siliques. As a loading control blots were probed with actin. Click here for file

Additional File 3 **Tissue-specific GUS activities under the control of the *AtOCT2 *Promoter**. In reproductive organs, pollen grains and stigma (A and B) as well as the veins of siliques and young siliques show GUS staining (C and D). Staining in leaves was present in cauline leaves (E) and rosette leaves (F). In mature leaves the signal is found in the vasculature (G), most probably in the phloem (K) whereas in younger leaves the vasculature was not stained, but the leaf blade was (H). Cross sections of young leaves show staining of the epidermis and parenchyma cells (I) and parenchyma cells around the phloem in older leaves (J). In roots the GUS signal was visible in two strands of the central cylinder, the initiation of lateral roots and at the root tips (L,M,N). Click here for file
