# Supplementary figures and images for: Stress regulated members of the plant organic cation transporter family are localized to the vacuolar membrane
Source: BMC Res Notes. 2008 Jul 11;1:43. doi: 10.1186/1756-0500-1-43 (PMC2519074; doi:10.1186/1756-0500-1-43)

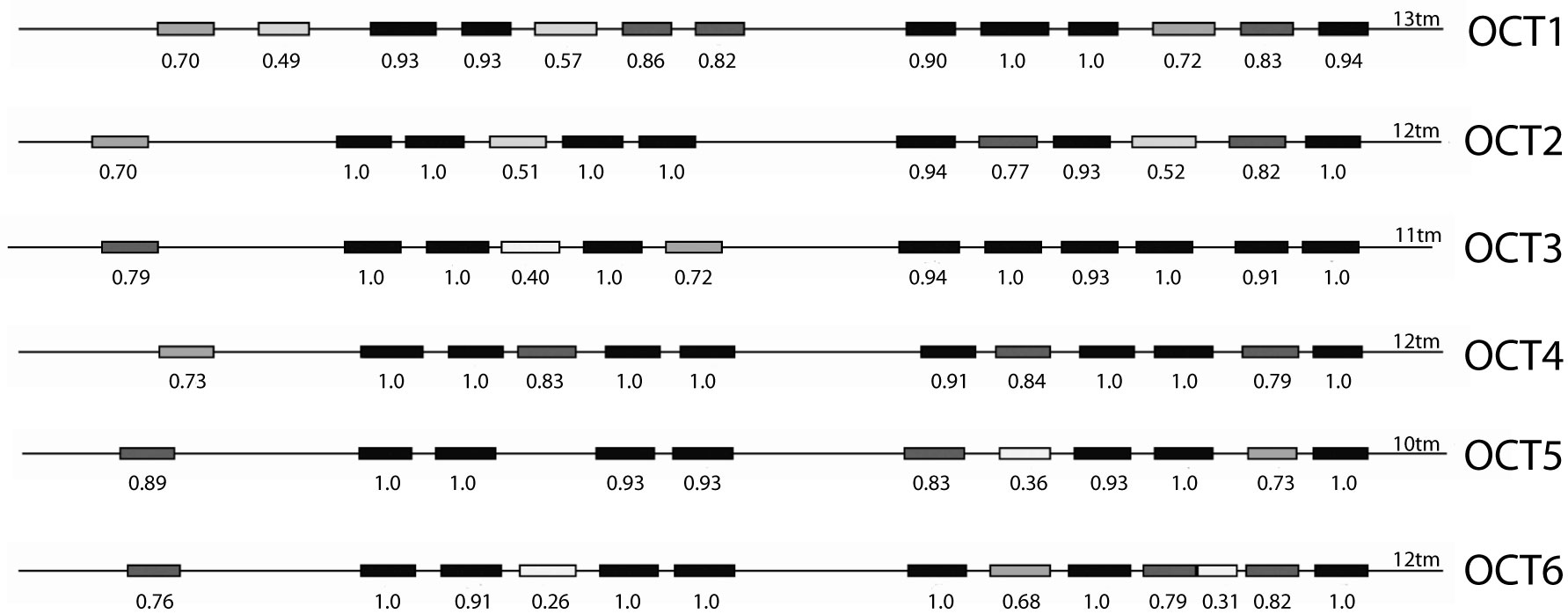

Supplement: Additional File 1 — Consensus prediction of transmembrane domains of the AtOCTs. Hydropathie analysis using the ARAMEMNON database [34] is based on the consensus of 11 prediction programmes. For AtOCT1 and AtOCT6 13 transmembrane domains are predicated, for AtOCT2, AtOCT3, and AtOCT4 12 transmembrane domains and for AtOCT5 11. Numbers score the probability of each predicted membrane domain of the AtOCTs. [file 1756-0500-1-43-S1.jpeg]

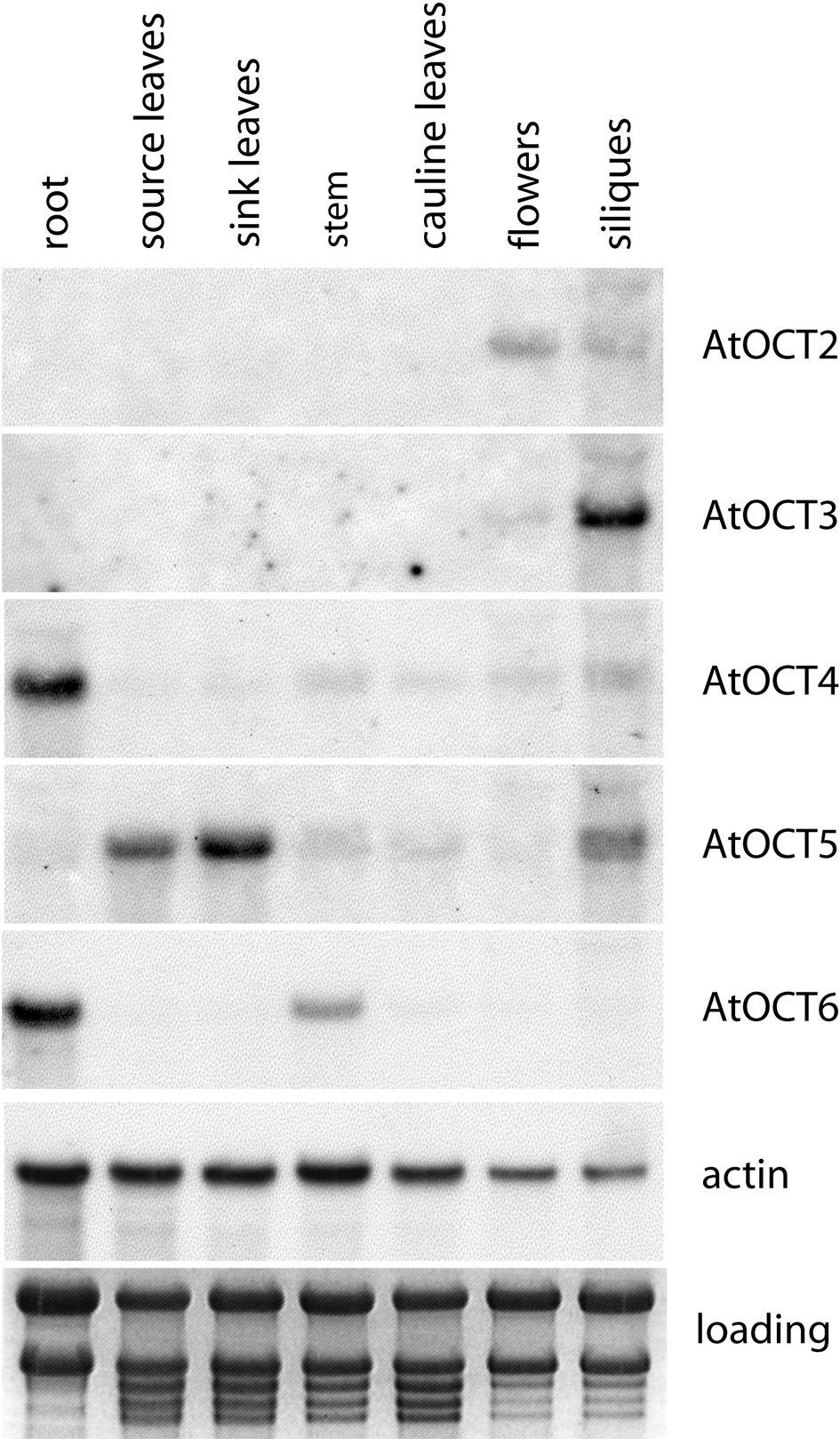

Supplement: Additional File 2 — Organ specific expression AtOCT2-AtOCT6 using RNA-Gel blot analysis. RNA-gel blot analysis showed a specific expression of the individual AtOCTs in Arabidopsis. AtOCT2 revealed a weak expression in flowers and siliques, AtOCT4 and AtOCT6 showed a predominant expression in roots, and AtOCT6 is also expressed in the stem. AtOCT 5 expression was strongest in sink leaves and weaker in mature leaves. AtOCT3 expression is strongest in siliques and a weak signal is detected in flowers, AtOCT2,3, and 5 also showed a weak signal in siliques. As a loading control blots were probed with actin. [file 1756-0500-1-43-S2.jpeg]

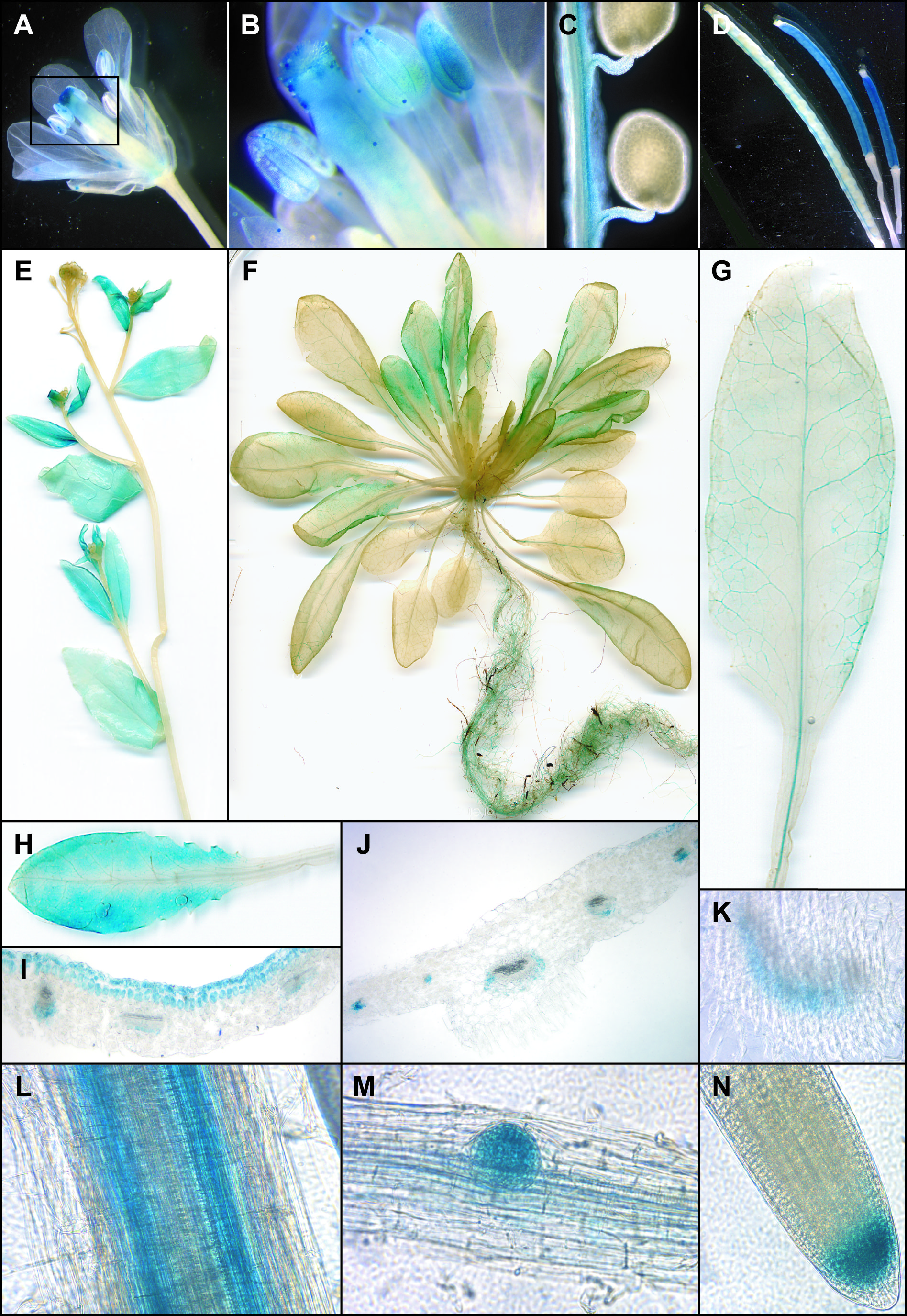

Supplement: Additional File 3 — Tissue-specific GUS activities under the control of the AtOCT2 Promoter. In reproductive organs, pollen grains and stigma (A and B) as well as the veins of siliques and young siliques show GUS staining (C and D). Staining in leaves was present in cauline leaves (E) and rosette leaves (F). In mature leaves the signal is found in the vasculature (G), most probably in the phloem (K) whereas in younger leaves the vasculature was not stained, but the leaf blade was (H). Cross sections of young leaves show staining of the epidermis and parenchyma cells (I) and parenchyma cells around the phloem in older leaves (J). In roots the GUS signal was visible in two strands of the central cylinder, the initiation of lateral roots and at the root tips (L,M,N). [file 1756-0500-1-43-S3.jpeg]
